# Quantum Coherence Control at Temperatures up to 1400 K

**DOI:** 10.1021/acs.nanolett.4c04359

**Published:** 2024-11-12

**Authors:** Jing-Wei Fan, Shuai-Wei Guo, Chao Lin, Ning Wang, Gang-Qin Liu, Quan Li, Ren-Bao Liu

**Affiliations:** † Department of Physics, The Chinese University of Hong Kong, Shatin, New Territories, Hong Kong, China; ‡ School of Physics, Hefei University of Technology, Hefei, Anhui 230601, China; § New Cornerstone Science Laboratory, The Chinese University of Hong Kong, Shatin, New Territories, Hong Kong, China; ∥ School of Physics, Hubei Key Laboratory of Gravitation and Quantum Physics, Institute for Quantum Science and Engineering, Huazhong University of Science and Technology, Wuhan, Hubei 430074, China; ⊥ Beijing National Laboratory for Condensed Matter Physics, Institute of Physics, Chinese Academy of Sciences, Beijing 100190, China; # Centre for Quantum Coherence, The Chinese University of Hong Kong, Shatin, New Territories, Hong Kong, China; ∇ The Hong Kong Institute of Quantum Information Science and Technology, The Chinese University of Hong Kong, Shatin, New Territories, Hong Kong, China

**Keywords:** High-temperature quantum control, quantum coherence, nitrogen-vacancy center, optically detected magnetic resonance

## Abstract

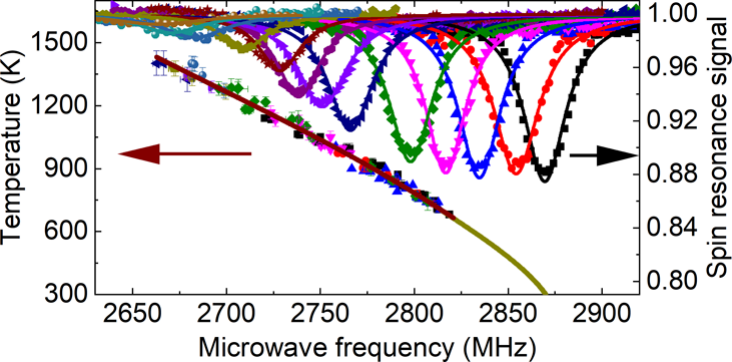

Coherent quantum control at high temperatures is important for expanding the quantum world and is useful for applying quantum technologies to realistic environments. Quantum control of spins in diamond has been demonstrated near 1000 K, with the spins polarized and read out at room temperature and controlled at elevated temperatures by rapid heating and cooling. Further increase of the working temperature is challenging due to fast spin relaxation in comparison with the heating and cooling rates. Here we significantly improve the heating and cooling rates by using reduced graphene oxide as the laser absorber and heat drain and hence realize coherent quantum operation at up to 1400 K, which is higher than the Curie temperatures of all known materials. This work facilitates the use of diamond sensors to study a wide range of magnetic effects in the high-temperature regime, such as thermoremanent magnetism and magnetic shape memory effects.

Maintaining and controlling quantum coherence usually requires well-isolated systems under stringent conditions such as low temperatures. Quantum coherence control at high temperatures is not only of interest to addressing the fundamental question about the boundary between the quantum and classical worlds but also useful for practical application such as quantum sensing of materials. As an outstanding example, quantum coherence of nitrogen-vacancy (NV) center spins in diamond^1^ can be prepared, detected, and controlled under ambient conditions,^2^ under high pressure,^3,4^ and in liquids.^5^ The continuous-wave optically detected magnetic resonance (ODMR) of NV spins persists up to a temperature of 700 K.^1^ Recently, using rapid heating and cooling of nanosystems, it has been demonstrated that NV spins in a nanodiamond (ND) can be first initialized at room temperature, then coherently controlled to perform quantum sensing such as nanomagnetometry at high temperature, and finally read out at room temperature. Such high-temperature quantum control and quantum sensing can facilitate the exploration of a broad range of thermal phenomena, such as magneto-structural transitions,^6,7^ thermoremanent magnetism in rock particles,^8^ heat-assisted magnetic recording,^9,10^ thermo-plasmonic of nanoparticles,^11,12^ and thermal management using two-dimensional materials.^13^

The previous research on high-temperature quantum coherence control is limited to about 1000 K,^14^ due to the rapid spin relaxation at high temperature (∼5 μs at about 1000 K), which is comparable to the heating and cooling times (∼2 μs). Achieving quantum coherence control at higher temperatures is not only desirable for a broader range of applications but may also enable in situ investigation of NV dynamics in NDs. Recent studies investigated thermal effects on spin properties and annealing behaviors of NV centers in bulk diamond.^15−17^ NV electron spins in bulk diamonds may exhibit longer spin relaxation and coherence times.^18,19^ However, we used nanodiamonds instead of bulk diamonds, because it is difficult to achieve rapid heating and cooling for bulk samples. Here, by largely improving the heating and cooling rates, we demonstrate quantum coherence control of NV electron spins in NDs at temperatures up to 1400 K, which, remarkably, is above the Curie temperatures of all known magnetic materials.

The optical setup is nearly the same as in our previous work,^14^ except that an air objective (NA = 0.85), an 850 nm laser (with maximum power on the sample around 12 mW), and a vacuum chamber were used in this work, as shown in Figure 1. The vacuum chamber was pumped by a mechanical pump combined with a turbomolecular pump to achieve a vacuum level at 5 × 10^–6^ Torr. rGO was used as the 850 nm laser heating material.

**Figure 1:**
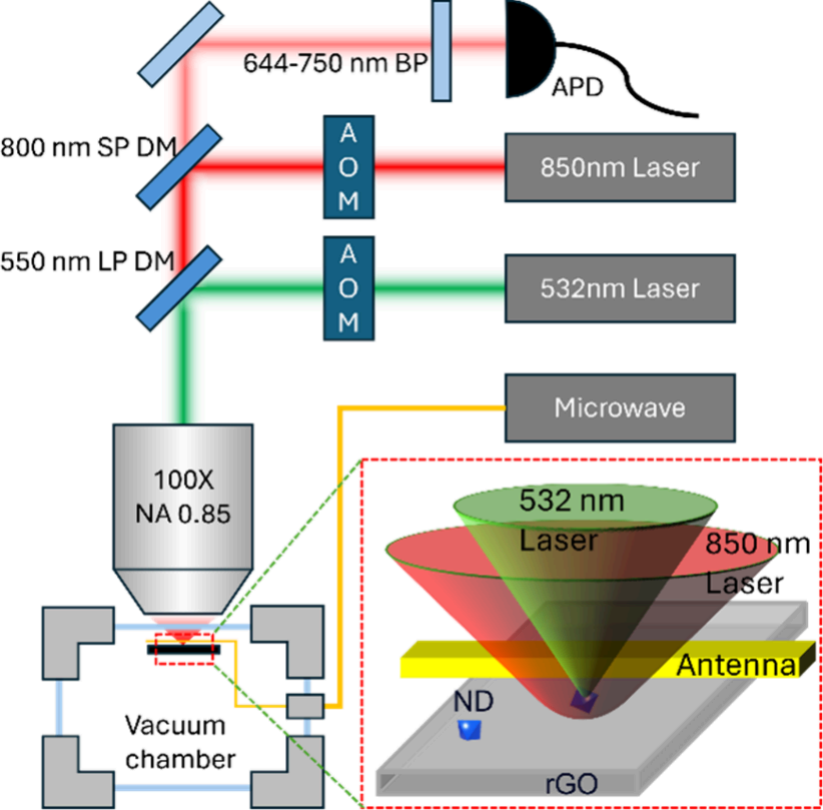
Schematic of the high-temperature ODMR setup. An 850 nm laser and a 532 nm laser are combined by an 800 nm short pass (SP) dichroic mirror (DM) and a 550 nm long pass (LP) DM. AOM: acousto-optic modulator. BP: bandpass filter. APD: avalanche photodiode. rGO: reduced graphene oxide.

Reading out spin states before they relax to thermal equilibrium requires fast heating and cooling. To increase the heating and cooling rates, we needed a substrate with a high photon-thermal conversion efficiency and a high heat conductivity. rGO is adopted for its excellent laser absorption^20^ and thermal conduction performance.^21^ We prepared rGO using a modified Hummer’s method^22^ and placed it on transmission electron microscopy (TEM) copper grids. We then dispersed NDs on the rGO film (Figure 1 and Figure 2a). We characterized the heating and cooling dynamics using the pulse sequence shown in Figure 2b. After optical polarization of the NV center spins, the ND was heated to a stationary temperature by a pulse of near-infrared (NIR, 850 nm) laser and then cooled down to room temperature for spin readout. A microwave pulse of 40 ns duration was applied for pulsed-ODMR measurement at different times in the heating and cooling stages. Figure 2c and 2d show the ODMR spectra of an ND on the rGO in the heating and cooling processes, respectively. We used Lorentzian fitting to determine zero-field splitting *D* and determined the corresponding temperatures using the temperature dependence of *D*.^1^ The temperature variation is shown in Figure 2e, with the heating and cooling time scales of approximately 233 (±5) and 243 (±12) ns, respectively. These heating and cooling times are shorter by about 1 order of magnitude than those (about 2 μs) previously achieved on amorphous carbon films in Ar atmosphere.^14^ This improvement overcomes the spin relaxation effect for achieving spin control at higher temperatures. Besides, the rGO exhibits excellent thermal stability in the vacuum chamber, as we did not observe a significant change in laser heating efficiency during the repeated heating/cooling experiments in about 5 months. Without the aging problem of amorphous carbon films, the use of an rGO facilitates long-term high-temperature ODMR (HiT-ODMR) experiments.

**Figure 2:**
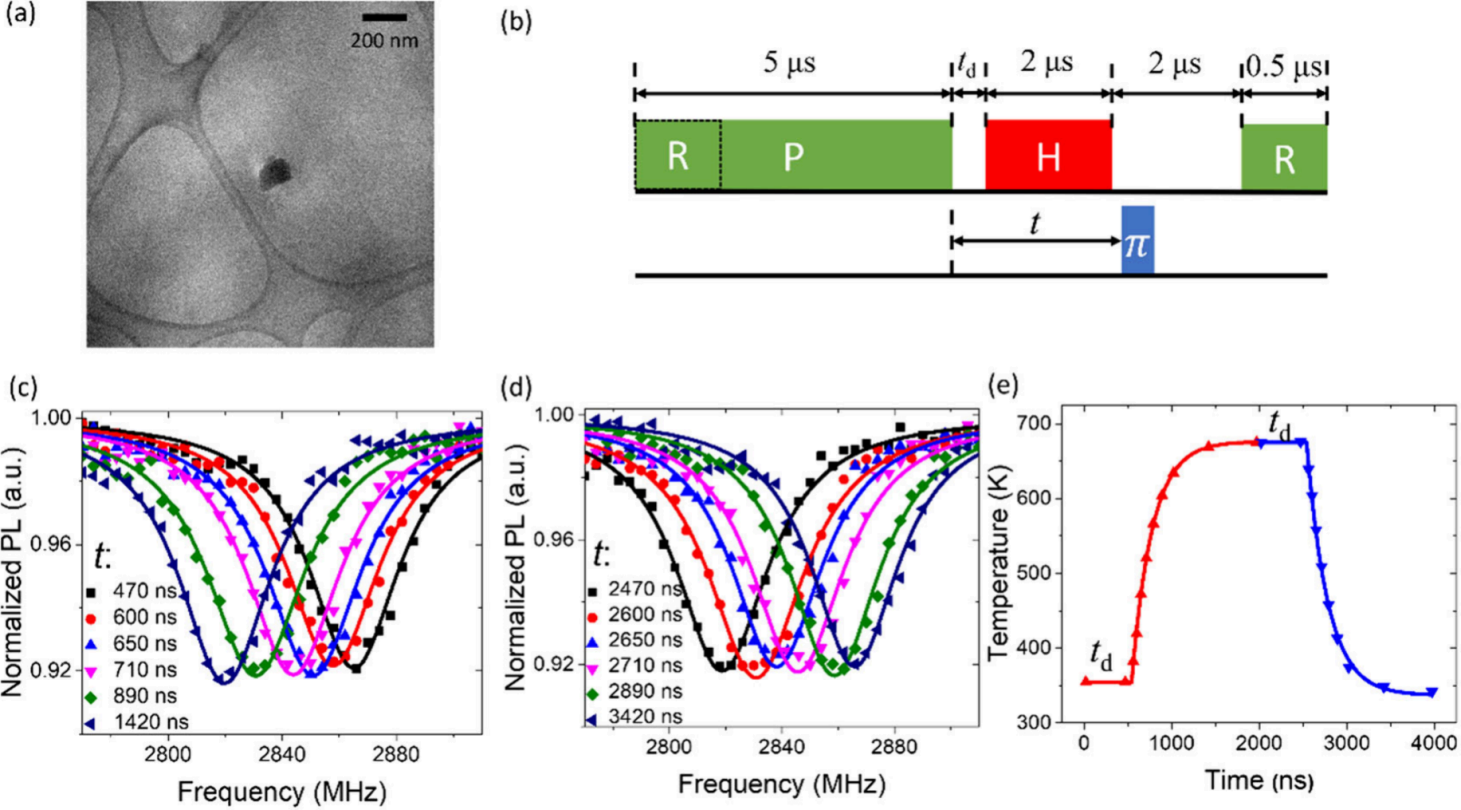
Heating and cooling dynamics of nanodiamonds on reduced graphene oxide. (a) TEM image of an ND located on rGO deposited on a copper grid with lacey carbon films. (b) Pulse sequence for characterizing the heating and cooling dynamics, including a 532 nm laser pulse with a duration of 5 μs for spin polarization (P) and readout (R), an 850 nm NIR laser pulse (τ_NIR_ = 2 μs) for heating (H), and a microwave π-pulse of 40 ns for spin control. The cooling time between H and R was set as 2000 ns. (c) and (d) ODMR spectra of an ND with the π pulse applied with *t* ranging (c) from 0 to 2000 ns (*t* ≤ τ_NIR_), covering the heating process, and (d) from 2000 to 4000 ns (*t* ≥ τ_NIR_), covering the cooling process. (e) Temperature as a function of time. The two flat stages (with duration *t*_d_) are due to the AOM delay in applying the NIR pulse and the time needed for heat diffusion from the heating spot to the ND.

To observe HiT-ODMR, we first polarized the NV center spins in an ND using a green laser pulse, then heated the ND using an NIR laser pulse, and at the end of the heating pulse applied a microwave pulse, which would flip the spins if it is resonant with the spin transition. After a cooling time of 2.5 μs, the spins were read out by using the fluorescence during the first 500 ns of the 532 nm laser pulse. Figure 3a shows the pulse sequence, and Figure 3b presents the ODMR spectra measured with various NIR laser powers for two typical NDs. The ODMR contrast decreases with increasing temperature. Nevertheless, the ODMR is still well observable at temperatures up to about 1429 (±11) K. The temperature dependence of the zero-field splitting *D* measured for eight NDs is shown in Figure 3c. We used the *D–T* relation in ref (1) to determine the temperature at the end of the heating pulse if *D* > 2815 MHz (corresponding to *T* < 700 K). The temperature >700 K was determined by extrapolation of the cooling curve, which was assumed to be an exponential function of cooling time. The *D*–*T* relation for 2760 MHz ≤ *D* ≤ 2815 MHz was calibrated first and then used to determine the *D*–*T* relation for 2700 MHz ≤ *D* ≤ 2760 MHz, which was in turn used to calibrate the *D*–*T* relation for 2663 MHz ≤ *D* ≤ 2700 MHz. This step-by-step calibration is to avoid extrapolation for temperatures far away from the range where the *D*–*T* relation is known (see the Supporting Information for details). The relation for *D* ≤ 2815 MHz is well fitted by *T* = 596119.636915 – 654.120036*D* + 0.241380*D*^2^ – 2.989338 × 10^–5^*D*^3^, or *D* = 2878.81872 + 0.00527*T* – 1.65597*T*^2^ + 3.9333 × 10^–8^*T*^3^. The reduction of contrast at high temperatures (see Figure 3d) can be ascribed to spin relaxation during heating and cooling processes, indicated by the good agreement between the measured contrast and the numerical simulation using a temperature-dependent spin relaxation rate (measured as shown in Figure 4; see the details of simulation in the Supporting Information).

**Figure 3:**
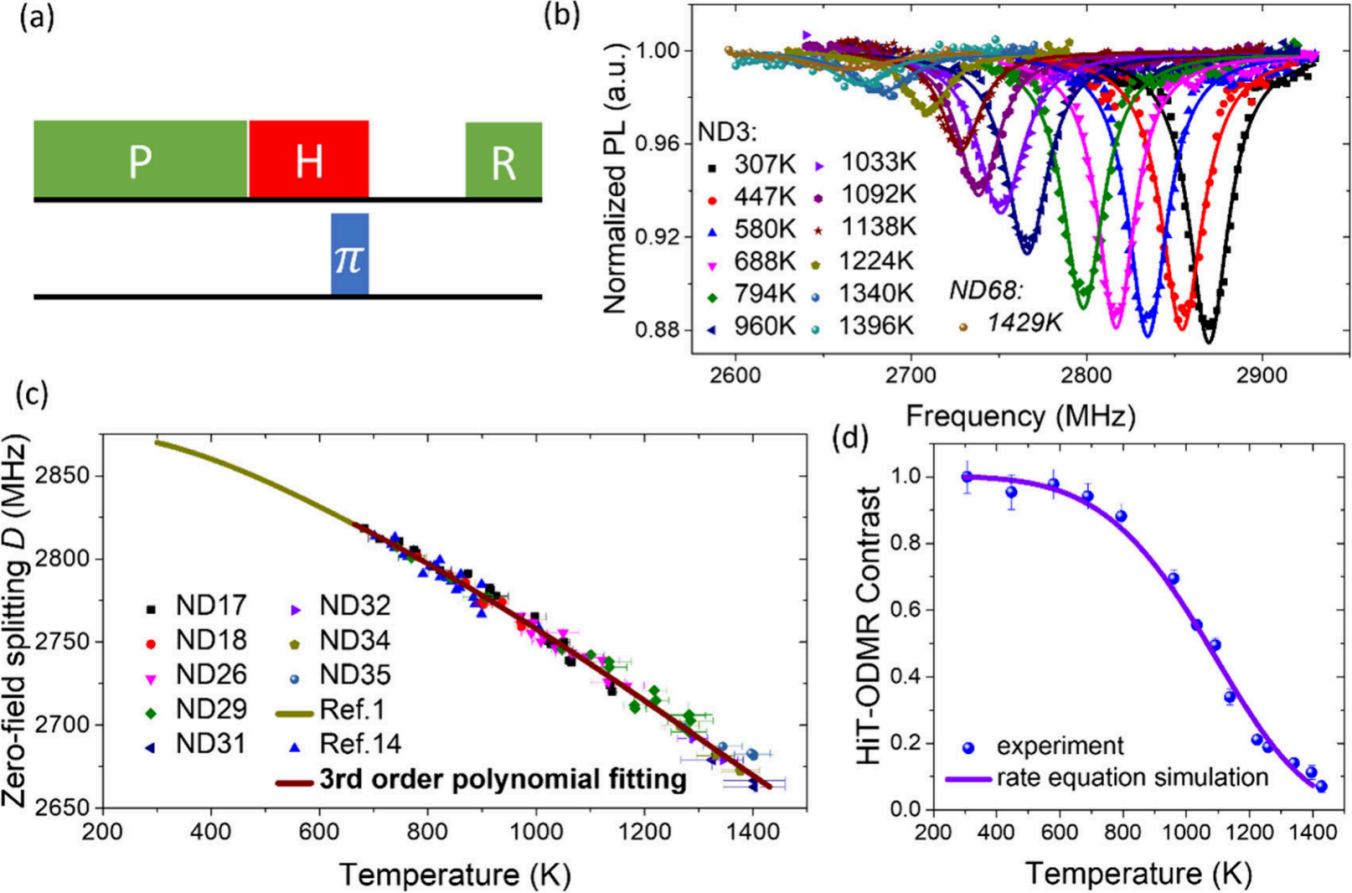
High-temperature ODMR of NV centers in NDs. (a) Pulse sequence for HiT-ODMR. The spins were polarized and read out by a green laser pulse (P) of duration 5 μs. The NIR heating pulse (H) had a duration of 2.5 μs. The cooling time was set as 2.5 μs. The NIR laser power was tuned to heat the NDs to various temperatures. A microwave pulse of duration 34 ns was applied at the end of the NIR pulse to measure ODMR. (b) ODMR spectra of two NDs (ND3 and ND68) for various temperatures. (c) The zero-field splitting *D* vs temperature *T* for eight NDs, in comparison with the fitting results in ref (1) and data points in ref (14). The brown curve is a third-order polynomial fitting of the *D–T* relation above 700 K. The error bars correspond to the uncertainties of extrapolation fitting the exponential cooling. (d) Contrast of the HiT-ODMR spectra measured at different temperatures (symbols) and numerical estimation obtained by solving the rate equation of spin relaxation (line).

**Figure 4:**
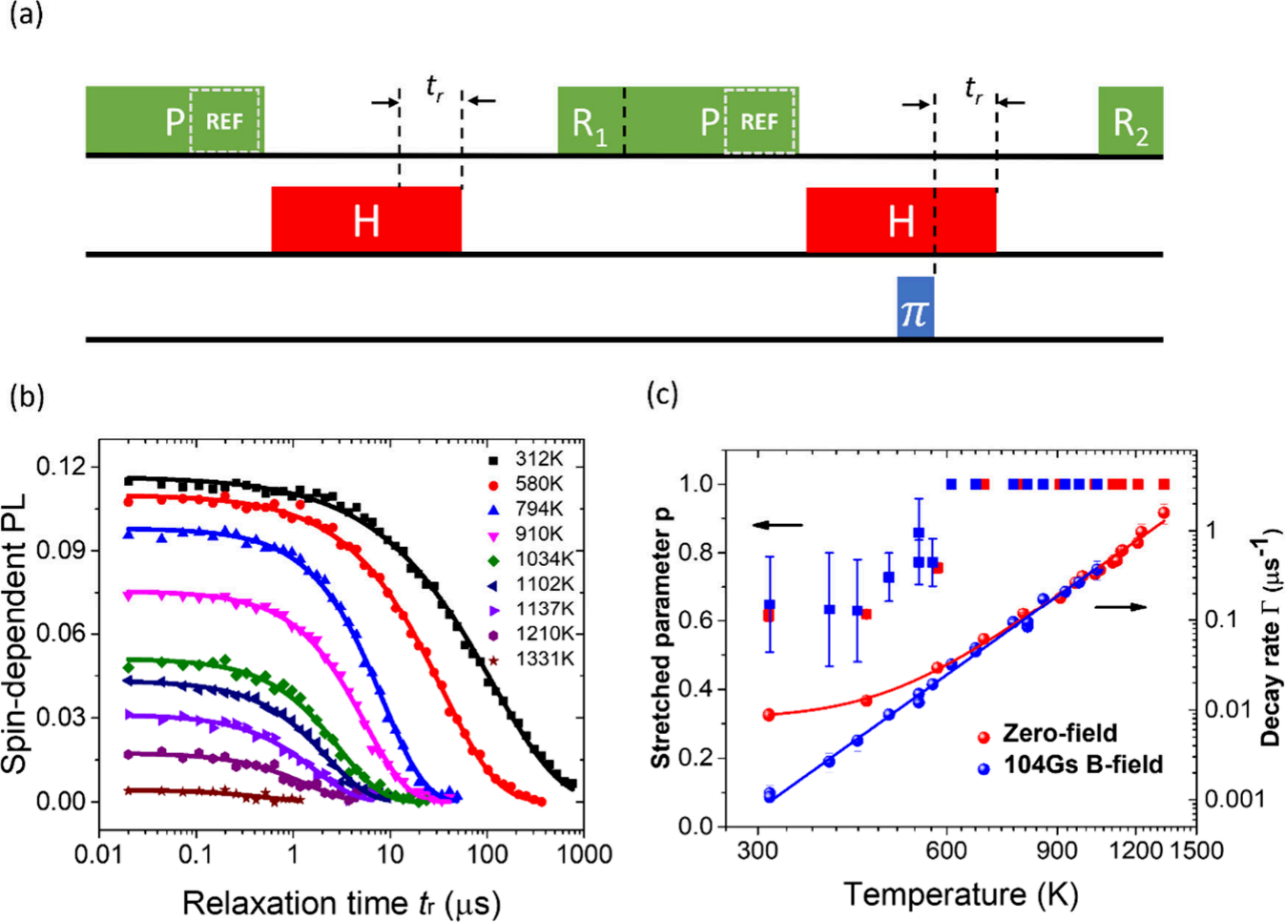
Temperature dependence of NV spin relaxation in a nanodiamond (ND3). (a) Two-unit pulse sequence for measuring the spin depolarization after a certain relaxation time (*t*_r_). Each unit is composed of a 5-μs green laser pulse applied at room temperature, then an NIR laser heating pulse (of duration 2.5 μs + *t*_r_, with or without a 34 ns microwave π pulse applied at 2.5 μs), and a fixed cooling period (2.5 μs) (after which the NV center spins are read out at room temperature, denoted as *R*_1/2_ in the green pulses). The difference between the photon counts with and without the microwave π pulse, normalized to the fluorescence recorded during a 500 ns period (denoted as REF in the green pulses), was measured as the spin polarization signal. (b) Spin polarization signal as a function of relaxation time *t*_r_ under near zero field for different temperatures (realized by using different NIR pulse powers). (c) Right axis: spin relaxation rate (1/*T*_1_) of NV spins in the ND as a function of temperature *T*. The solid red circles were measured without applying a magnetic field and fitted by *T*_1_^–1^ = Γ_0_ + *A* × *T*^5^ with Γ_0_ = 7.94 (±0.42) × 10^–3^ μs^–1^ and *A* = 3.063 (±0.084) × 10^–16^ μs^–1^ K^–5^. The solid blue circles were measured with a 104 G magnetic field applied to lift the degeneracy between the four crystallographic NV orientations. The blue circles were fitted by *T*_1_^–1^ = *A* × *T*^5^ with *A* = 3.157 (±0.103) × 10^–16^ μs^–1^ K^–5^. The *T*_1_ relaxation curves were fitted by a stretched exponential decay function  with *p* being the stretching exponent. Blue/red squares are the stretching exponent *p* as a function of *T* with/without the magnetic field (axis on the left). The error bars are standard fitting errors.

Spin relaxation is the main factor that limits the HiT-ODMR contrast and sets the ultimate limit of the spin coherence time, which, in turn, determines the magnetometry sensitivity. Cross-relaxation and spin–lattice relaxation are two main mechanisms of spin relaxation, with the former relying on the degeneracy of spin transition frequencies^23^ and the latter being temperature dependent. To measure the spin relaxation rate at high temperature, we used a two-unit pulse sequence as shown in Figure 4a. Figure 4b plots the spin polarization signal (under a zero magnetic field) as a function of relaxation time for various temperatures. The relaxation time at room temperature was 120 μs and could be elongated to 930 μs by applying a magnetic field of 104 G to lift the spin resonance degeneracy of 4 crystallographic NV orientations (see Figure 4c and Figure S1). This degeneracy dependence suggests the existence of cross-relaxation in the ND, which may occur through spin diffusion^24^ and polarization transfer to non-NV spins on the surface of the ND. In the range of 300–600 K, the relaxation curves were fitted by a stretched exponential decay function , where the stretching exponent *p* is about 0.6 near room temperature and increases to 1 as temperature approaches 600 K (see Figure 3c). The reason that the stretching exponent deviates from 1 could be due to the inhomogeneity of the spin relaxation time of NV centers with different locations in the ND.^23,25^ With a magnetic field applied to suppress the cross-relaxation, the spin relaxation rates follow the *T*^5^ dependence. Above 600 K, either under zero-field or a magnetic field, the relaxation curves could be well fitted by an exponential decay , suggesting that the phonon scattering dominates the spin relaxation. The *T*^5^ dependence, resulting from two-phonon Raman processes,^26^ appears to be valid up to 1400 K.

To demonstrate quantum coherence control at high temperatures, we performed Rabi oscillation of the NV center spins in NDs using the pulse sequence shown in Figure 5a. The heating NIR laser was turned on for 3.5 μs + *t*_MW_. In the first unit, a microwave pulse of duration *t*_MW_ was applied after 3.5 μs (when the temperature reached its stationary value). The second unit without a microwave pulse was used as a reference to exclude effects not related to the spin states. Figure 5b shows the Rabi oscillations at different temperatures. The visibility of the Rabi oscillation decreases with increasing temperature, which can be attributed to the effect of spin relaxation during the heating and cooling processes. The decay time of Rabi oscillation, *T*_1ρ_, decreases significantly when the temperature exceeds 800 K (see Figure 5d). We measured the free-induction decay time *T*_2_^*^ (which is inversely proportional to the inhomogeneous broadening; see Figure 5c and measurement details in the SI) and also found the decrease of *T*_2_^*^ as the temperature exceeded 800 K. We estimated the value of *T*_1ρ_ based on the free-induction decay time *T*_2_^*^ by numerical simulation (details in the SI) and found good agreement (see Figure 5d). Therefore, the decrease in Rabi oscillation lifetime can be attributed to the inhomogeneous broadening. In the previous report, the *T*_2_^*^ of a single NV center and ensemble NV centers in bulk are independent of temperature below 625 K.^1,27^ Here we show that *T*_2_^*^ of ensemble NV spins in nanodiamonds is still independent of temperature up to around 800 K. A possible cause of the increase of inhomogeneous broadening at temperatures above 800 K is temperature fluctuations induced by thermal drift and laser power fluctuation, but further study is needed to unambiguously determine the causes. Despite the decrease in visibility and lifetime, we succeeded in detecting Rabi oscillations of NV spins up to 1280 K.

**Figure 5:**
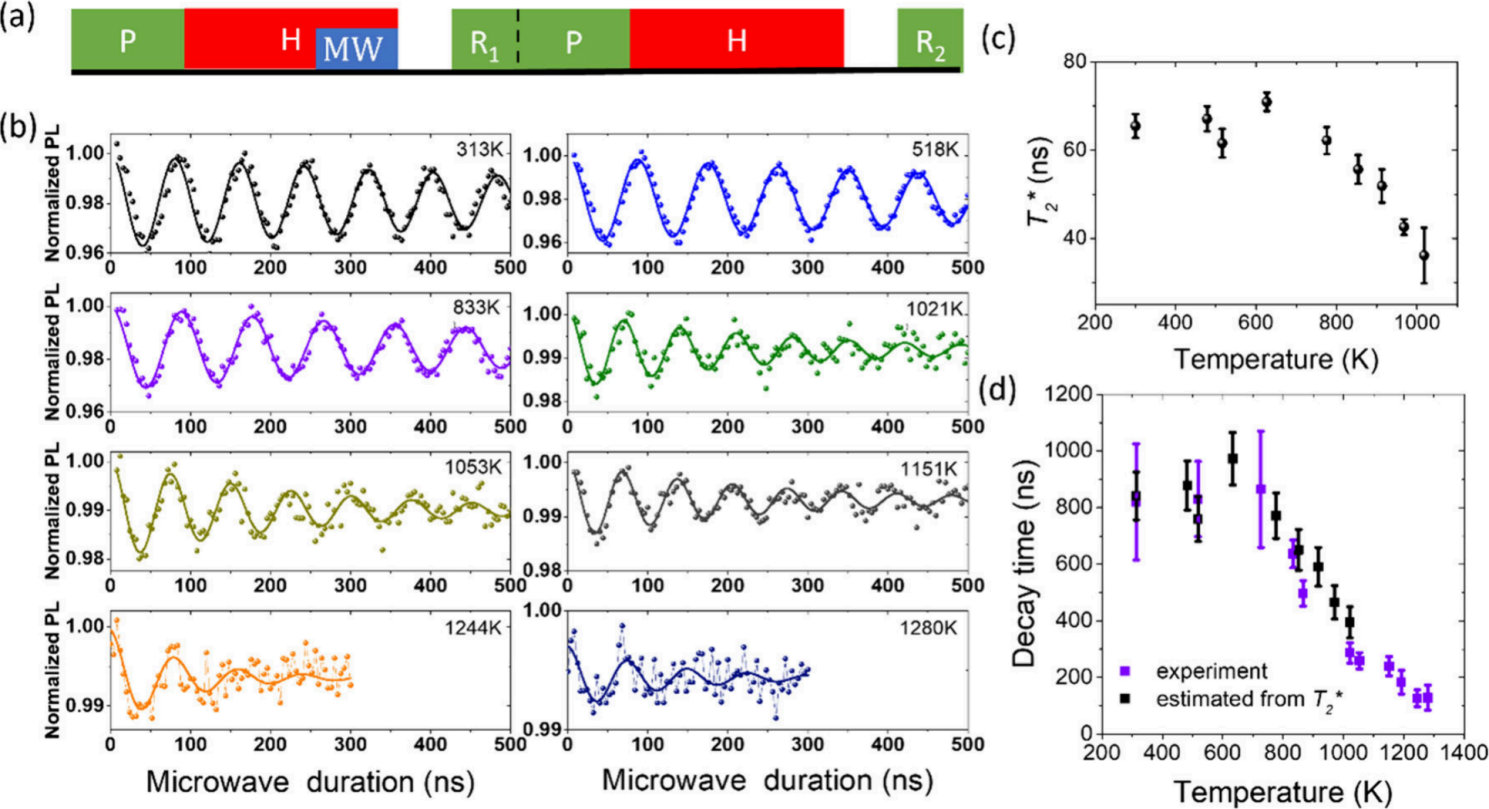
Rabi oscillations of NV center spins at high temperatures. (a) Pulse sequence. In the first/second unit, the signal/reference photocount *R*_1/2_ was taken during the first 0.5 μs of the 5 μs 532 nm laser pulse with/without a microwave pulse (MW) applied at the latter stage of the heating pulse (H). (b) Normalized photon counts (*R*_1_/*R*_2_) as functions of the microwave pulse duration, showing Rabi oscillations of NV center spins. The data for temperatures below 1200 K were collected from ND3, and those above 1200 K were from ND61. An external magnetic field (103 G) was applied to lift the degeneracy of the 4 crystallographic NV orientations, and the microwave pulse was set resonant with the NV centers that had the largest splitting (i.e., were best aligned with the magnetic field). (c) Free-induction decay time *T*_2_^*^ of *m*_s_ = 0 to *m*_s_ = +1 transition as a function of temperature. (d) Decay time of Rabi oscillation from experiments and estimated from *T*_2_^*^ as functions of temperature. The error bar is from fitting error.

We managed to apply spin flips and hence measure the spin resonances of NV centers at temperatures above 1400 K. This high temperature is above the Curie temperatures of all known magnetic materials. With the extended operating temperature range, nanoscale resolution, multimodal sensing, and fast heating/cooling dynamics, our scheme extends the application of NV-based sensing to the study of a broad range of effects, including the in situ investigation of annealing effects on NV centers, the ancient thermoremanent magnetic field recorded in rock particles, and the magnetic phase transition dynamics.

Fast spin relaxation is the main limitation to observe and control quantum coherence at even higher temperatures. It is possible to use microdiamonds to realize ODMR at even higher temperatures, since microdiamonds have longer spin relaxation times^28^ and are still small enough for rapid heating and cooling. NV centers hosted in high quality diamond (such as diamond nanopillars) may exhibit long memory time in their nuclear spins and thus enable spin coherence signal above 1400 K. Optimization on heating and cooling methods (including speed, stability, and reproducibility) may further improve the maximum quantum coherence control temperature of NV center spins.

## Supporting Information Available

Samples, rate equation simulation, effects of magnetic field on relaxation time *T*_1_, methods of temperature calibration, temperature dependence of *T*_2_^*^, and simulation on the effect of inhomogeneous broadening on the decay time of Rabi oscillation (PDF)
