# Supplementary material for: Quantum Coherence Control at Temperatures up to 1400 K
Source: Nano Lett. 2024 Nov 12;24(46):14806–11. doi: 10.1021/acs.nanolett.4c04359 (PMC11583336; doi:10.1021/acs.nanolett.4c04359)
Supplement: Supplementary file 1 — nl4c04359_si_001.pdf [file nl4c04359_si_001.pdf]

# Supplementary Information for

# Quantum coherence control at temperatures up to 1400 K

Jing-Wei Fan[1,2,#], Shuai-Wei Guo[1,3,#], Chao Lin[1], Ning Wang[1,4], Gang-Qin Liu[5], Quan Li[1,6,7 †], Ren-Bao Liu[1,3,6,7 *]

1. Department of Physics, The Chinese University of Hong Kong, Shatin, New Territories, Hong Kong, China
2. School of Physics, Hefei University of Technology, Hefei, Anhui 230601, China
3. New Cornerstone Science Laboratory, The Chinese University of Hong Kong, Shatin, New Territories, Hong Kong, China
4. School of Physics, Hubei Key Laboratory of Gravitation and Quantum Physics, Institute for Quantum Science and Engineering, Huazhong University of Science and Technology, Wuhan, Hubei 430074, China
5. Beijing National Laboratory for Condensed Matter Physics, Institute of Physics, Chinese Academy of Sciences, Beijing 100190, China
6. Centre for Quantum Coherence, The Chinese University of Hong Kong, Shatin, New Territories, Hong Kong, China
7. The Hong Kong Institute of Quantum Information Science and Technology, The Chinese University of Hong Kong, Shatin, New Territories, Hong Kong, China

# These authors contribute equally.

†liquan@cuhk.edu.hk

* rbliu@cuhk.edu.hk

**Note 1: Samples.**

**Samples.** NDs (average size ~120 nm) were purchased from Adamas Nanotechnologies. Each contains about 1200 NV centers with concentration of nitrogen about 200 ppm. Reduced graphene oxide was prepared via a modified Hummers' method, as described in [1]. We dropped rGO on TEM copper grids purchased from TED Pella and then dispersed NDs on rGO. The NV spin properties (including ODMR, $T_1$ and $T_2^*$) measured with rGO in this work are similar to those measured without rGO in our previous work [2].

**Note 2: Rate equation simulation for HiT-ODMR contrast.**

The rate equation of spin contrast can be expressed by

$$\frac{dC(t)}{dt} = -\Gamma C(t),$$

where $C(t)$ is the spin contrast at time *t*, $\Gamma = 1/T_1$ is the spin relaxation rate, which is temperature dependent as given by the fitting formula of Fig. 3c in main text. During the heating and cooling processes, the temperature profile $T(t)$ is described by the following two equations, respectively.

During heating: $T(t) = -(T_s - T_o)\exp\left(-\frac{t}{t_1}\right) + T_s,$

During cooling: $T(t) = (T_s - T_o\ )\exp\left(-\frac{t}{t_1}\right) + T_o,$

where $T_s$ is the saturated temperature, $T_o$ is environment temperature, and $t_1$ is the heating/cooling time scale. For ND3, we measured $t_1$=529 ns.

The solution of the spin contrast at the end of the heating and cooling process is

$$C(t) = C(t=0)\exp\left(-\int_0^{t_c}\Gamma dt\right)\exp\left(-\int_0^{t_h}\Gamma dt\right),$$

where $t_h$ is the time duration for heating and $t_c$ is the time duration for cooling. In the HiT-ODMR experiments, $t_h = t_c = 2.5\ \mu s$ were used. Taking the temperature dependent $\Gamma$ and time dependent temperature into the equation, we estimated the values of HiT-ODMR contrast at different saturated temperatures [see Fig. 2(d) in main text].

**Note 3: Effects of magnetic field on relaxation time $T_1$**

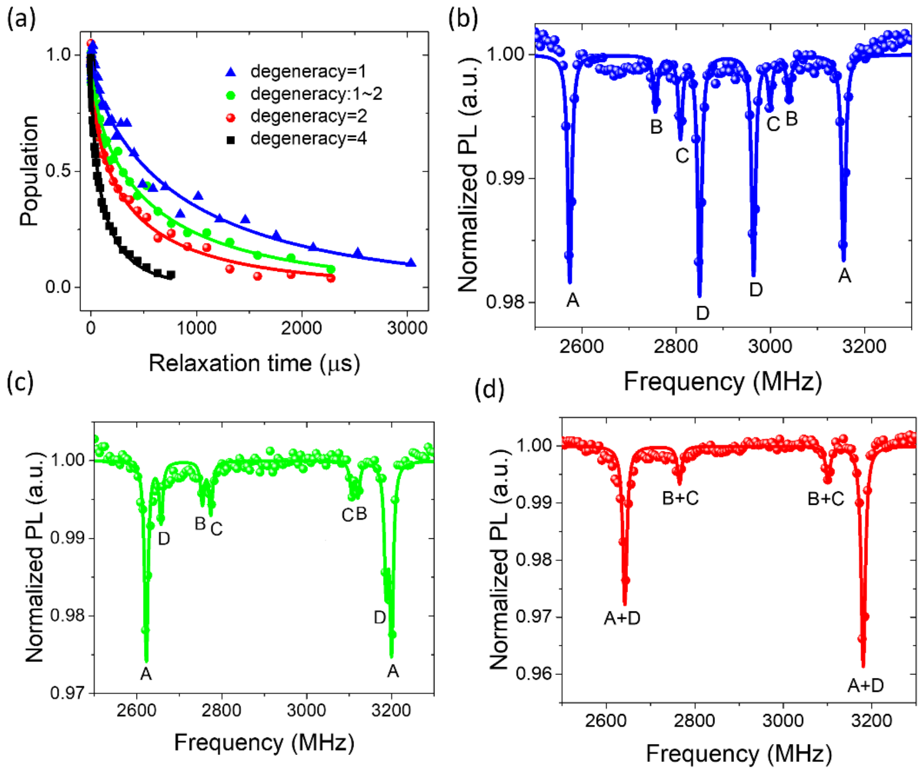

Fig. S1 (a) $T_1$ relaxation of NV spins with different degeneracy in a nanodiamond (ND3) at room temperature. Here, the degeneracy represents the number of crystallographic orientations of NV centers that have similar resonance frequencies (with difference less than the strength of NV-NV spin interaction). By manipulating external magnetic field, we got different degeneracy degrees: (b) degeneracy = 1, where the corresponding magnetic field was used for $T_1$ measurement in main text; (c) degeneracy: 1~2 (which indeicates that the cross-relaxation is not fully suppressed with a 12.8 MHz splitting between A and D peaks due to the relatively strong dipolar interaction between a portion of NV center that have small distances). (d) degeneracy = 2. Degeneracy = 4 is obtained under zero filed. A, B, C, D stand for ODMR dips contributed from NV centers of 4 different crystallographic orientations. The rightest peaks of the ODMR spectra were used for measurement of $T_1$ relaxation time. $T_1$ relaxation time increases with the decrease of degeneracy, indicating the suppression of cross-relaxation.

**Note 4: Methods of temperature calibration.**

Temperature calibration was used to determine the *D*-*T* relation for temperatures above 700 K. The temperature < 700 K was determined by the *D*-*T* relation given in [3]. Therefore, a part of a cooling curve (starting from temperatures around 700 K) is

known. The temperature > 700 K can be determined by extrapolation of the cooling curve, which is assumed to be an exponential function of cooling time. The ZFS $D$ obtained from the ODMR spectrum with microwave pulse applied at a specific time point corresponds to the temperature determined by the extrapolation for the same time point. Thus, the $D$-$T$ relation with temperatures above 700 K is obtained.

Based on the method, we measured cooling curves of nanodiamonds to calibrate the $D$-$T$ relation. The time $t = 0$ was set as the starting point of cooling, excluding the time delay $t_d$ due to AOM delay in applying the NIR pulse and the time needed for heat diffusion from the heating spot to the ND. The time delay $t_d$ was determined by fitting a cooling process that started from a temperature below 700 K with a flat curve followed by an exponential cooling curve. Curve I in Fig. S2a shows the fitting curve we used to determine $t_d$. Hollow circles in curves II and III of Fig. S2a represents the deduced temperatures by extrapolation of the cooling curves. Fig. S2b-d show the ODMR spectra obtained by applying microwave at time points shown in curves I-III in Fig. S2a, respectively. The ZFS $D$ determined from the ODMR spectrum and the deduced temperature at a same time point give the $D$-$T$ relation for 2760 MHz$\leq D \leq$2815 MHz (shown in Fig. S3), which agrees well with previous work [2]. The $D$-$T$ relation for 2760 MHz$\leq D \leq$2815 MHz was fitted and then used to deduce $D$-$T$ relation for 2700 MHz $\leq D \leq$ 2760 MHz, as shown by Fig. S4-5. The $D$-$T$ relation for 2700 MHz $\leq$

$D \leq$ 2760 MHz was in turn used to deduce the *D*-*T* relation for 2663 MHz $\leq D \leq$ 2700 MHz (see Fig. S6). Finally, we obtained the *D*-*T* relation for 2663 MHz $\leq D \leq$ 2815 MHz and fitted it with a 3$^{rd}$ order polynomial formula described in main text. For Fig. 2b, Fig. 2d, Fig.3 and Fig. 4 in the main text, temperatures above 700K were determined by the formula.

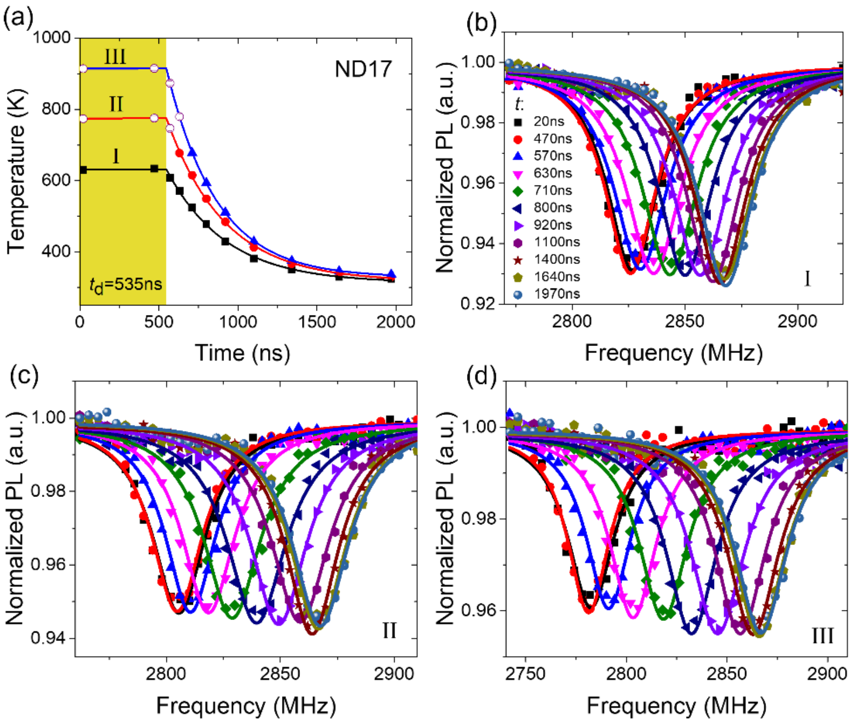


Fig. S2 (a) Cooling curves starting from various temperatures of a nanodiamond (ND17) as a function of time *t*. Solid symbols are for temperatures below 700K (*D* > 2815 MHz), which were determined by the *D*-*T* relation in [3]. For curves II and III, the temperatures during the flat stage and immediately after the heating pulse (open symbols) were determined by extrapolating the cooling curves from below 700 K (solid symbols). The error bars correspond to the uncertainties of extrapolation fitting the

exponential cooling. (b-d) ODMR spectra obtained by applying microwave pulse at time points in curves I-III, correspondingly.

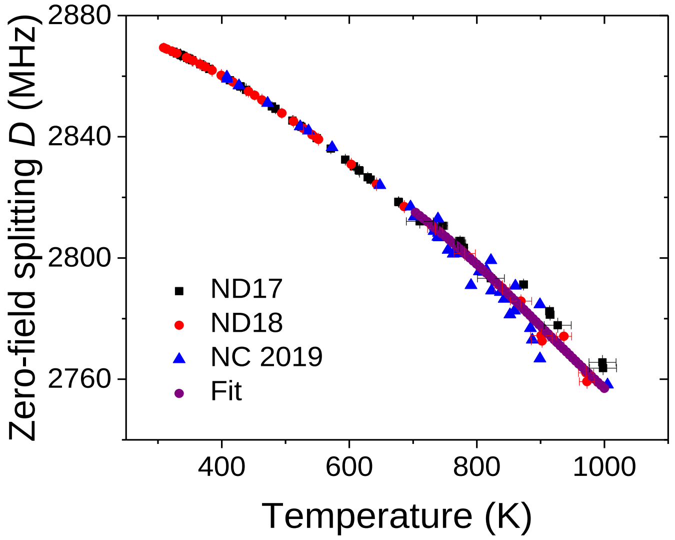


Fig. S3 *D*-*T* relation for 2760 MHz $\leq D \leq$ 2815 MHz, calibrated with two nanodiamonds (ND17 and ND18). The error bars correspond to the uncertainties of extrapolation fitting the exponential cooling. Calibrated results in [2] are also plotted here, denoted as NC_2019. The relation is fitted with a formula $T = 7090795.9855571 - 7649.4179047633 \times D + 2.7527387858147 \times D^2 - 3.3040764833069 \times 10^{-4} \times D^3$.

.

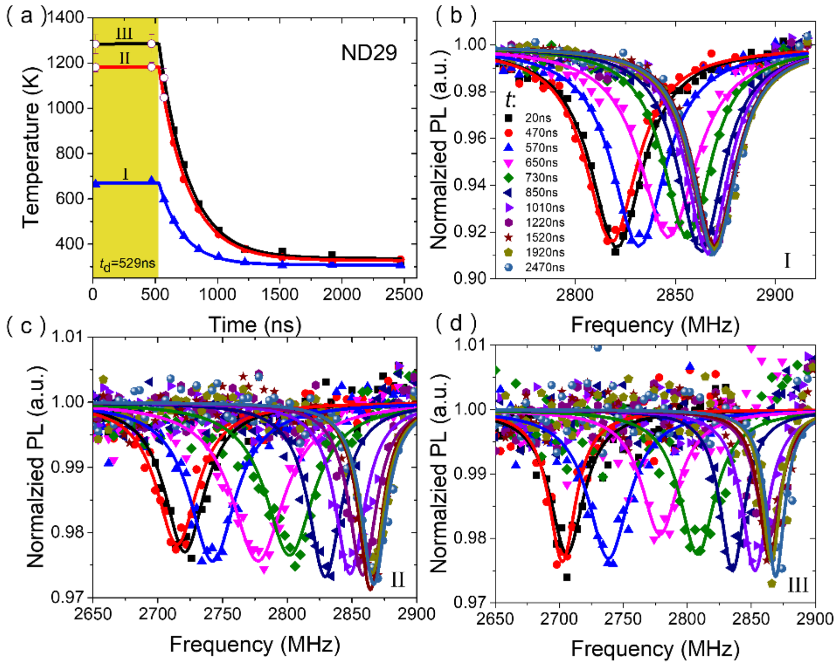


Fig. S4 (a) Cooling curves starting from various temperatures of a nanodiamond (ND29) as a function of time *t*. Solid symbols are for temperatures corresponding to $D \geq$ 2760 MHz. In curves II and III, the temperatures during the flat stage and immediately after the heating pulse (open symbols) were determined by extrapolating the cooling curves from below 1000 K (solid symbols, determined from the *D*-*T* relation in Fig. S3). The error bars correspond to the uncertainties of extrapolation fitting the exponential cooling. (b-d) ODMR spectra obtained by applying microwave pulse at time points in curves I-III, correspondingly.

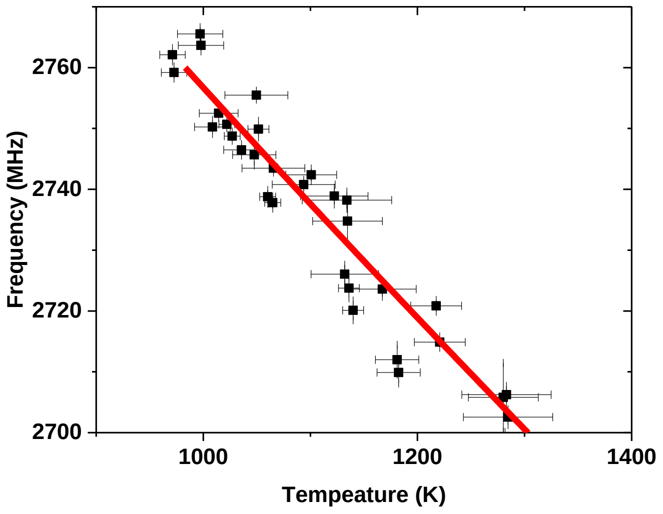


Fig. S5 $D$-$T$ relation for 2700 MHz $\leq D \leq$2760 MHz. The symbols show calibrated results from NDs and the red line shows fitting formula: $T = 37800.168062112 - 21.523024636337 \times D + 0.002965034496269 \times D^2$. The error bars correspond to the uncertainties of extrapolation fitting the exponential cooling.

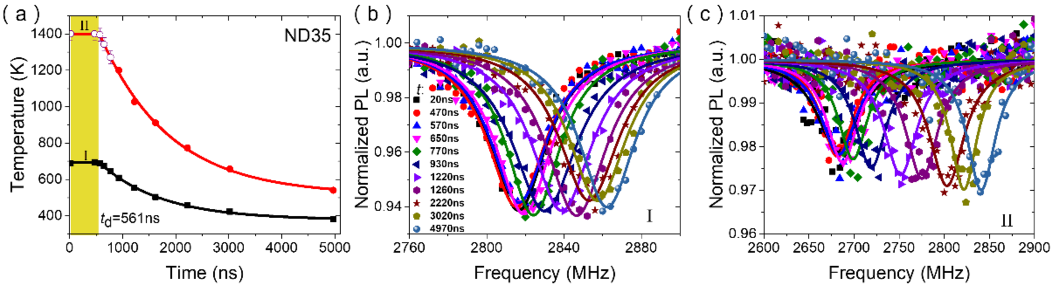


Fig. S6 (a) Cooling curves starting from various temperatures of a nanodiamond (ND35) as a function of time $t$. Solid symbols are for temperatures corresponding to $D \geq$ 2700 MHz. In curve II, the temperatures during the flat stage and immediately after the heating pulse (open symbols) were determined by extrapolating the cooling curves from

below 1200 K (solid symbols, determined by the *D*-*T* relation in Figs. S3 and S5). The error bars correspond to the uncertainties of extrapolation fitting the exponential cooling. (b) and (c) ODMR spectra obtained by applying microwave pulse at time points in curves I and II, respectively.

We tried Raman spectroscopy of rGO to determine the temperature independently. However, the Raman peak was too broad [1] and did not yield satisfactory precision of temperature measurement.

**Note 5:** Temperature dependence of $T_2^*$.

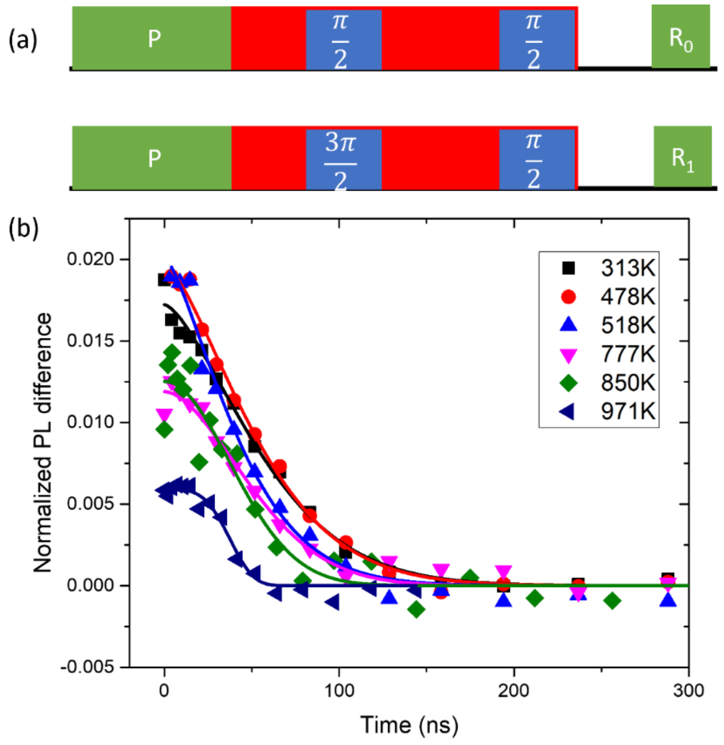


Fig. S7 (a) Pulse sequence for the measurement of free-induction decay time $T_2^*$ at high temperature. (b) Decay of the coherence between the NV spin states with $m_s = 0$

and $m_s = +1$ measured at various temperatures. An external magnetic field (about 103 Gauss) was applied to lift the degeneracy of the 4 crystallographic NV orientations, and the microwave pulse was set resonant with the NV centers that had the largest splitting (i.e., were best aligned with the magnetic field).

As shown in Fig. S7a, the sequence used for measurement of free-induction decay time $T_2^*$ consists of two units. First, the NV center spins were polarized by a 5 µs green laser pulse, and then the nanodiamond was heated to a stable temperature with a 3.5 µs NIR laser pulse. We applied a π/2 microwave pulse and then maintained the temperature for various times, then applied another π/2 pulse, and recorded the photon counts ($R_0$) after a cooling process of 3.5 µs. The second unit was the same as the first, except that the first π/2 pulse was replaced by a 3π/2 pulse. The difference in photocounts $R_1$ and $R_0$, normalized by their sum is recorded as the spin coherence signal. In this way, we obtained the decay curve and fitted it by $y = Ae^{-(t/T_2^*)^p}$, as shown Fig. S7b. The measured $T_2^*$ of the NV spins is shown in main text Fig. 4c.

**Note 6: Simulation on the effect of inhomogeneous broadening on the decay time of Rabi oscillation.**

We conducted numerical simulation to estimate the effects of inhomogeneous broadening on the decay time of Rabi oscillations, $T_{1\rho}$. The inhomogeneous

broadening is assumed to have a Gaussian distribution of $f$. The spin population in the state $m_s = 0$ under the Rabi oscillation averaged over the detuning $\delta \equiv f - f_{\mathrm{NV}}$ (with $f_{\mathrm{NV}}$ being the resonance frequency of the NV center spins) is

$$P_0(t) = \int \frac{\omega_R}{2\sqrt{\omega_R{}^2 + \delta^2}} \left[1 + \cos\left(2\pi\sqrt{\omega_R{}^2 + \delta^2}t\right)\right] g(\delta) d\delta,$$

where $\omega_R$ is Rabi frequency of the microwave pulse, $t$ is microwave duration, and the Gaussian function $g(\delta) = \frac{1}{\sqrt{2\pi}\sigma}\exp(-\frac{\delta^2}{2\sigma^2})$ has the standard deviation $\sigma = 1/(\sqrt{2}\pi T_2^*)$. The Rabi frequency $\omega_R$ was obtained from the fitting of experimental data of Rabi oscillation at room temperature. With this method, we obtained the oscillation signal at various microwave durations and fitted it with $P_0(t) = A(\ \exp\left(-\frac{\mathrm{t}}{T_{1\rho}}\right)\cos(2\pi\omega t) + 1)$, in which $A$, $T_{1\rho}$, $\omega$ are fitting parameters. The obtained $T_{1\rho}$ agrees well with experimental results, as shown in main text Fig. 4d, indicating that inhomogeneous broadening is the main reason for the variation of Rabi oscillation decay time with elevated temperature.

## References


[1] Lin, C.; Niu, C.; Xu, X.; Li, K.; Cai, Z.; Zhang, Y.; Wang, X.; Qu, L.; Xu, Y.; Mai, L., A facile synthesis of three dimensional graphene sponge composited with sulfur nanoparticles for flexible Li–S cathodes. Physical Chemistry Chemical Physics 2016, 18 (32), 22146-22153.

[2] Liu, G.-Q.; Feng, X.; Wang, N.; Li, Q.; Liu, R.-B., Coherent quantum control of nitrogen-vacancy center spins near 1000 kelvin. Nature Communications 2019, 10 (1), 1344.

[3] Toyli, D. M.; Christle, D. J.; Alkauskas, A.; Buckley, B. B.; Van de Walle, C. G.; Awschalom, D. D., Measurement and Control of Single Nitrogen-Vacancy Center Spins above 600 K. Phys. Rev. X 2012, 2 (3), 031001.
